# Successful management of penetrating shrapnel injury to the left ureter with delayed projectile migration through the urinary tract

**DOI:** 10.1002/iju5.12623

**Published:** 2023-08-17

**Authors:** Roman Gutvert, Artem Kobirnichenko, Yevhen Bidula, Vasyl Balabanyk

**Affiliations:** ^1^ Urology Clinic of National Military Medical Clinical Center Kyiv Ukraine

**Keywords:** combat, gunshot trauma, ureter, ureteral injury, ureteral injury, war in Ukraine

## Abstract

**Introduction:**

We present a case of successful endoscopic treatment of a penetrating shrapnel injury to the left ureter. The patient experienced spontaneous migration of the projectile through the urinary tract, leading to renal colic.

**Case presentation:**

A military man sustained multiple shrapnel injuries to the soft tissues and abdomen, including lacerations of the intestines, during enemy shelling in eastern Ukraine. On the 35th day after the trauma, the patient reported sudden pain in the left lumbar area. Subsequent investigation confirmed the presence of a foreign body in the lower part of the left ureter, accompanied by contrast extravasation. Ureteroscopy revealed a round‐shaped metal shrapnel, which was removed. A double‐J stent was inserted into the left ureter.

**Conclusion:**

The endoscopic extraction of the shrapnel fragment and the subsequent placement of a ureteral stent for the repair of the ureteral injury have proven to be safe and effective methods.

**Abbreviations & Acronyms**
CT: computed tomography
IV: intravenous

**Keynote message**

In patients with previous gunshot injuries to the abdomen, the migration of retained projectiles should be considered as a potential cause for renal colic. Furthermore, the presence of shrapnel fragments within the ureter, leading to obstruction, should prompt consideration for their endoscopic removal.

## Introduction

The ongoing conflict in Ukraine has witnessed the frequent use of multiple‐launch rocket systems, cruise missiles, and other high‐energy weapons, resulting in severe injuries among military personnel. These injuries include abdominal wounds, vascular damage, limb amputations, as well as genitourinary trauma.^1^ Ureteral trauma is relatively rare and accounts for less than 1% of both blunt and penetrating genitourinary injuries.^2^ Gunshot wounds are the most common cause of ureteral injuries, with the proximal ureter being the most frequently affected site.^3^ Such injuries often occur in conjunction with damage to abdominal and pelvic organs.^4^, ^5^, ^6^

The management of wounds primarily depends on the location and severity of the injury. In this context, we present a case report of a successful endoscopic treatment for a penetrating shrapnel injury to the left ureter. The patient experienced the subsequent spontaneous migration of the projectile through the urinary tract, resulting in renal colic.

## Case presentation

A 46‐year‐old military man sustained multiple shrapnel injuries to the soft tissues of the face, chest, hands, and legs, along with a penetrating abdominal injury and lacerations of the intestines during enemy shelling in the Donbass region. In the initial surgical treatment at a mobile field hospital, a laparotomy was performed to explore the peritoneal cavity, suturing the intestinal lacerations, and conducting primary surgical debridement of the soft tissue wounds. No retroperitoneal injury was detected during laparotomy. The immediate post‐operative period was uneventful, with the patient receiving pain medication and broad‐spectrum antibiotics. The abdominal wall wound and numerous smaller soft tissue wounds healed through primary intention. Subsequently, the patient was transferred to a civilian hospital for further non‐surgical treatment and rehabilitation.

Sequential survey X‐rays and CT scans were conducted, revealing multiple round‐shaped fragments with metal density in the soft tissues of the abdominal wall, retroperitoneal fat, pelvis, and both thighs. Superficially located shrapnel fragments were removed under local anesthesia, while deeper fragments were left in place.

**Fig. 1:**
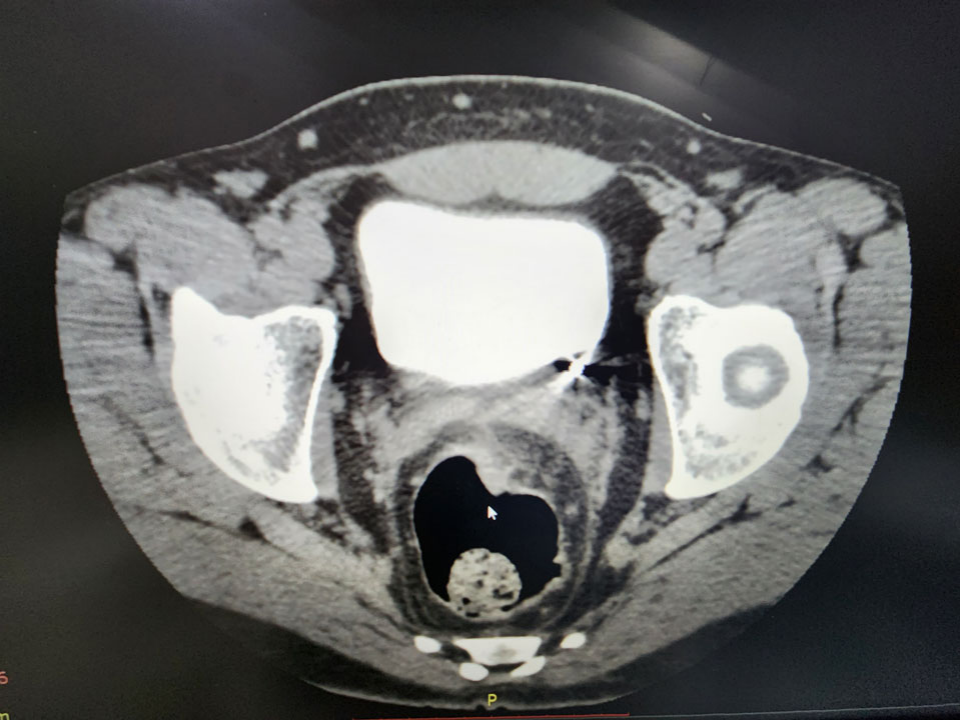
CT of the lower abdomen and pelvis shows a well‐defined metal fragment in the left ureter close to the urinary bladder.

**Fig. 2:**
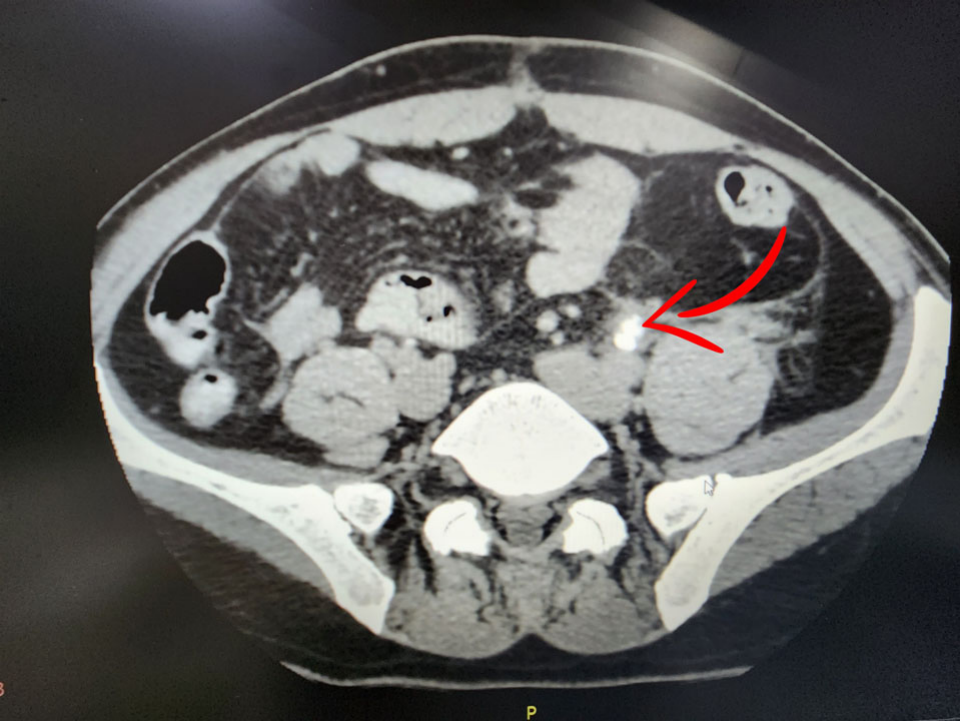
Extravasation of contrast at the level of middle third of the left ureter (red arrow).

On the 35th day after the trauma, the patient, who had been relatively well, complained of sudden paroxysmal pain in the left lumbar area, radiating to the groin, accompanied by frequent urination and weakness. An ultrasound examination revealed dilatation of the collective system of the left kidney and the proximal part of the left ureter. Subsequent CT scan with IV contrast enhancement confirmed impaired left kidney function due to the presence of a metal foreign body in the lower part of the left ureter (Fig. 1). Additionally, minimal contrast extravasation was observed at the level of the middle third of the left ureter (Fig. 2). Laboratory results showed an elevated serum creatinine level of 148 μmol/L and the presence of red blood cells in the urinalysis.

**Fig. 3:**
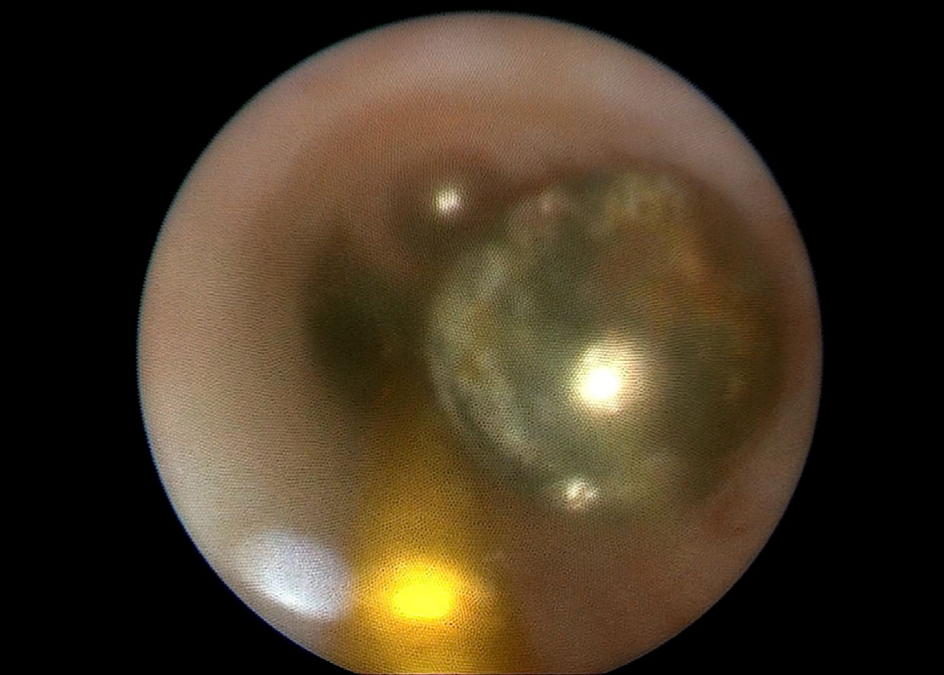
Ureteroscopy. Metal shrapnel before entrapment by helical stone extractor.

**Fig. 4:**
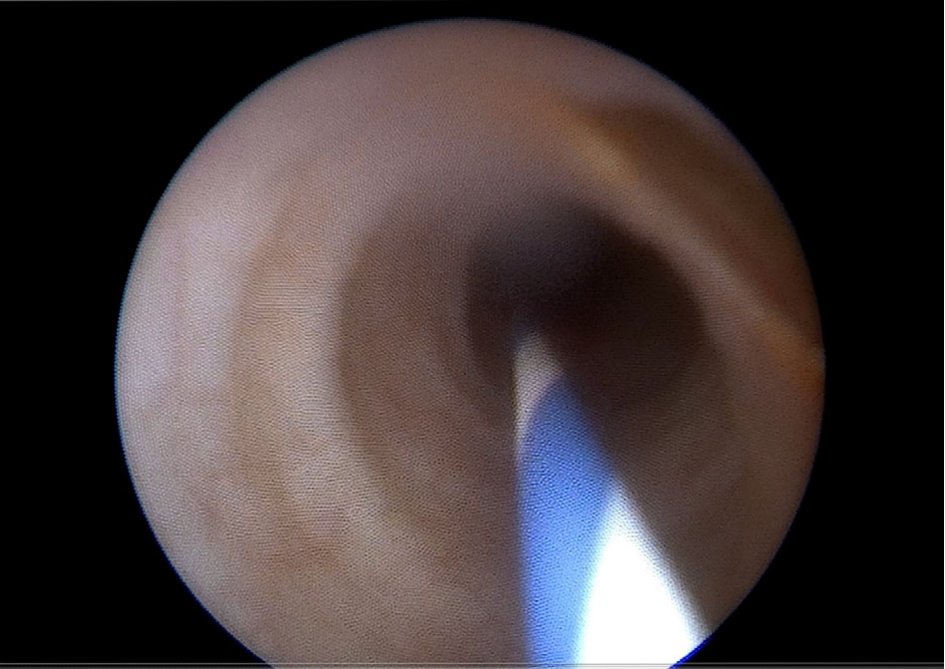
Ureteroscopy. Defect at the antero‐lateral wall of the middle third of the left ureter (upper right part of the figure).

After a brief preparation, which involved prescribing painkillers (dexketoprofen 50 mg), antispasmodics (drotaverine 80 mg), an antibiotic (levofloxacin 500 mg), and proton pump inhibitors (omeprazole 40 mg), the patient was brought to the operating room. Under spinal anesthesia, a rigid ureteroscope Karl Storz (Tuttlingen, Germany) was inserted through the urethra and urinary bladder, reaching the left ureteral orifice. In the lower third of the left ureter, a 4 mm round‐shaped metal shrapnel, partially encrusted and brown in color, was found (Fig. 3). The shrapnel was safely extracted using a 3.2Fr captura helical stone extractor (Cook Medical, Bloomington, IN, USA). Subsequent examination revealed a small 6 × 7 mm defect on the anterolateral wall of the middle third of the left ureter (Fig. 4). No other pathologies or traumatic defects were identified during a thorough examination. After removing the ureteroscope, a double‐J 5Fr stent (Cook Medical, Bloomington, IN, USA) was inserted into the left ureter. The correct positioning of the stent was confirmed through survey X‐ray.

The postoperative period proceeded without any complications. A follow‐up ultrasonography performed 3 days later revealed no signs of fluid in the pelvis or left flank, and there was no dilatation observed in the collective system of the left kidney. The stent was successfully removed after 1 month. Due to small size of contrast extravasation at the site of ureter injury (less than 1 cm according to initial CT scan), no ureterography or ureteroscopy were considered to be reasonable after stent removal. Ultrasound examination of the left kidney revealed no signs of hydronephrosis after 3 months.

## Discussion

Timely diagnosis of the shrapnel rupture of the left ureter in this case was challenging due to objective difficulties. These included limited accessibility to diagnostic resources such as CT at the front‐line hospital, the absence of typical clinical presentation due to the small size of the ureteral rupture, and the lack of urine leakage possibly due to the projectile itself obstructing the perforation defect. However, the spontaneous migration of the shrapnel within the urinary tract eventually caused symptoms resembling renal colic, leading to appropriate assessment and successful management.

It is important to note that while small fragments may pass through the ureter spontaneously, large projectiles can result in “shrapnel colic” if they traverse the ureter. In this particular instance, a survey abdominal X‐ray with IV contrast would have confirmed the diagnosis and potentially prevented complications such as the formation of urinoma or pyelonephritis.

Ureteroscopy with lithotripsy or lithoextraction is a well‐established procedure for managing ureteral stones during peaceful times. It should be considered as a viable option for cases involving ureteral obstruction caused by migrated projectiles, as it is a relatively straightforward procedure when performed by experienced practitioners, with favorable post‐operative recovery outcomes.

## Conclusion

Our findings demonstrate the safety and effectiveness of utilizing rigid ureteroscopy for the extraction of shrapnel fragments and the placement of ureteral stents to repair incomplete proximal ureteral injuries. This procedure, when performed by experienced surgeons, has shown promising results and may offer a new indication for its use. Maintaining a high index of suspicion is crucial in order to promptly identify potential lesions, minimize complications, and initiate immediate treatment.

## Author contributions

Roman Gutvert: Conceptualization; data curation; formal analysis. Artem Kobirnichenko: Data curation; investigation; visualization; writing – original draft. Yevhen Bidula: Investigation; methodology; project administration. Vasyl Balabanyk: Project administration; supervision; writing – review and editing.

## Conflict of interest

The authors declare no conflict of interest.

## Approval of the research protocol by an Institutional Reviewer Board

The ethics committees of National Military Medical Clinical Center gave approval for this study (#5/23) and written informed consent was obtained in accordance with the World Medical Association Helsinki Declaration.

## Informed consent

Written informed consent was obtained from the patient for publication of this article and accompanying images.

## Registry and the Registration No. of the study/trial

Not applicable.
